# Mucocutaneous Manifestations in Kidney Transplant Patients: Risk Factors

**DOI:** 10.7759/cureus.36177

**Published:** 2023-03-15

**Authors:** Yasmina El Arabi, Fouzia Hali, Yasmine Mahdar, Sophia Zahid, Mohamed Ali Zamd, Soumiya Chiheb, Benyounes Ramdani

**Affiliations:** 1 Dermatology, Ibn Rochd University Hospital, Casablanca, MAR; 2 Nephrology, Ibn Rochd University Hospital, Casablanca, MAR

**Keywords:** dermatological complications, muco-cutaneous diseases, • kidney transplantation, kidney transplant recipients, immuno suppressant

## Abstract

Introduction

Mucocutaneous complications in kidney transplant patients are due to drug toxicity or immunosuppression. The main objective of our study was to determine the risk factors associated with their occurrence.

Methods

We conducted a prospective analytical study (January 2020- June 2021) including kidney transplant patients seen at the Nephrology Department. We described the characteristics of the patients who presented mucocutaneous complications and then compared them to those who didn’t to deduce the risk factors. Statistical analysis was performed using SPSS 20.0 (p<0.05).

Results

Of the 86 patients recruited, thirty patients had mucocutaneous complications. The mean age was 42.73, with a male predominance (73%). Ten kidney transplants were performed from a living-related donor. All the patients received corticosteroids, Mycophenolate Mofetil, and the Calcineurin Inhibitor: Tacrolimus (76.7%) or Ciclosporin (23.3%). Induction was performed with Thymoglobulin (n=20) or Basiliximab (n=10). Mucocutaneous complications were dominated by infectious manifestations (53.4%): eight cases of fungal infections; six cases of viral infections: warts (n=3), herpes labialis (n=2), intercostal herpes zoster (n=1), and two cases of bacterial infections: atypical mycobacteria and boils. Inflammatory complications (36.6%) included acne (n=4), urticaria (n=3), rosacea (n=1), simple maculopapular exanthema (n=1), aphthous lesion (n=1), and black hairy tongue (n=1). Actinic keratosis, skin xerosis, and bruises were found in one patient respectively. The evolution with a symptomatic treatment was good in all the patients. After statistical analysis, the factors significantly associated with the occurrence of mucocutaneous complications were advanced age, male gender, anemia, HLA non-identical donor, as well as the use of Tacrolimus or Thymoglobulin.

Conclusion

Infectious mucocutaneous complications are the most common dermatological manifestations among renal transplant recipients. Their occurrence is related to advanced age, male gender, anemia, HLA non-identical donor, and the use of Tacrolimus or Thymoglobulin.

## Introduction

Kidney transplantation is the most common solid organ transplantation [1]. It is the treatment of choice for end-stage chronic renal failure and allows a significant gain in healthy life expectancy [2]. These kidney transplants would not have been possible without the introduction of immunosuppressants to prevent organ rejection. However, their use isn't without risks. They can lead to many complications either by direct effect or due to immunosuppression caused by the blockage of lymphocyte reactions against micro-organisms and tumor cells [3]. While general systemic complications in kidney transplant recipients have often been reported, dermatological ones have rarely been described. Chronic use of immunosuppressants after transplantation alters immunity and puts these patients at an increased risk of mucocutaneous complications, including drug side effects, immune-mediated effects of the transplanted organ, opportunistic infections, and malignancy. Immunosuppression required to maintain allograft function in the recipient results in impaired cell-mediated immunity, making these patients prone to various mucocutaneous infections. A recent review of 134 kidney transplant recipients showed that 78% of patients developed mucocutaneous infections, most commonly dermatomycosis, shingles, and folliculitis. These infections can be a primary focus or dissemination of a systemic infection. Immunosuppressive drugs can also potentiate the effects of other carcinogens, such as ultraviolet radiation that causes premalignant lesions and skin carcinoma [4]. The objective of our study was to determine the risk factors associated with the occurrence of mucocutaneous complications in kidney transplant patients.

## Materials and methods

We conducted a prospective analytical study including renal transplant patients who attended the Ibn Rochd University Hospital, Renal Transplantation Unit, Casablanca, Morocco, for routine checkup examinations between January 2020 and June 2021. We considered two groups of patients: group 1 including patients who presented mucocutaneous complications (36 patients) and group 2 including patients who didn't have mucocutaneous complications (50 patients). The two groups matched for age and median age of renal transplantation. The data were collected using an analysis grid. Informed consent was obtained from all subjects. Each patient was examined by the same dermatologist looking for the presence of skin, mucous membrane, hair, and nail disorders. The investigation also included blood examination, fungal/bacterial cultures, and histopathologic examination when indicated. Dermatologic findings were classified as mucocutaneous infections (bacterial, fungal, and viral), inflammatory lesions, and pre-cancerous and cancerous lesions.

The statistical analysis was conducted with SPSS (Statistical Package for the Social Sciences) software (version 20.0). The effects of quantitative variables (age and median age of renal transplantation) on the appearance of mucocutaneous disorders were evaluated using the Student’s t-test, and that of qualitative variables (gender, diabetes, arterial hypertension, heart disease, obesity, anemia, type of Human Leukocyte Antigen, and the use of Tacrolimus, Ciclosporin, Thymoglobulin or Basiliximab) using the chi-square test. A p-value of less than 0.05 was considered significant.

## Results

Among the 86 patients recruited during our study period, thirty patients presented mucocutaneous complications (34.88%). The mean age was 42.73 years (standard deviation= 13.006) with a clear male predominance (73%) and a sex ratio M/F of 2.75 (22M/8F). The majority were phototype III-IV. Diabetes and/or obesity were noted in eight patients, arterial hypertension in 50 patients, and heart disease in four patients. Fifteen patients had anemia.

The median age of renal transplantation was four years [1-11 years]. Transplantation was from an HLA identical related living donor in ten patients. The causative nephropathies of end-stage chronic renal failure were dominated by indeterminate nephropathy in half of the patients, followed by hypertensive nephroangiosclerosis in seven patients (23.4%), and glomerular or malformative nephropathy in four patients (13.3%). All the patients received oral corticotherapy, Mycophenolate Mofetil, and the Calcineurin inhibitor: Tacrolimus (n=23) or Ciclosporin (n=7). Induction was performed with Thymoglobulin (n=20) or Basiliximab (n=10).

**Figure 1:**
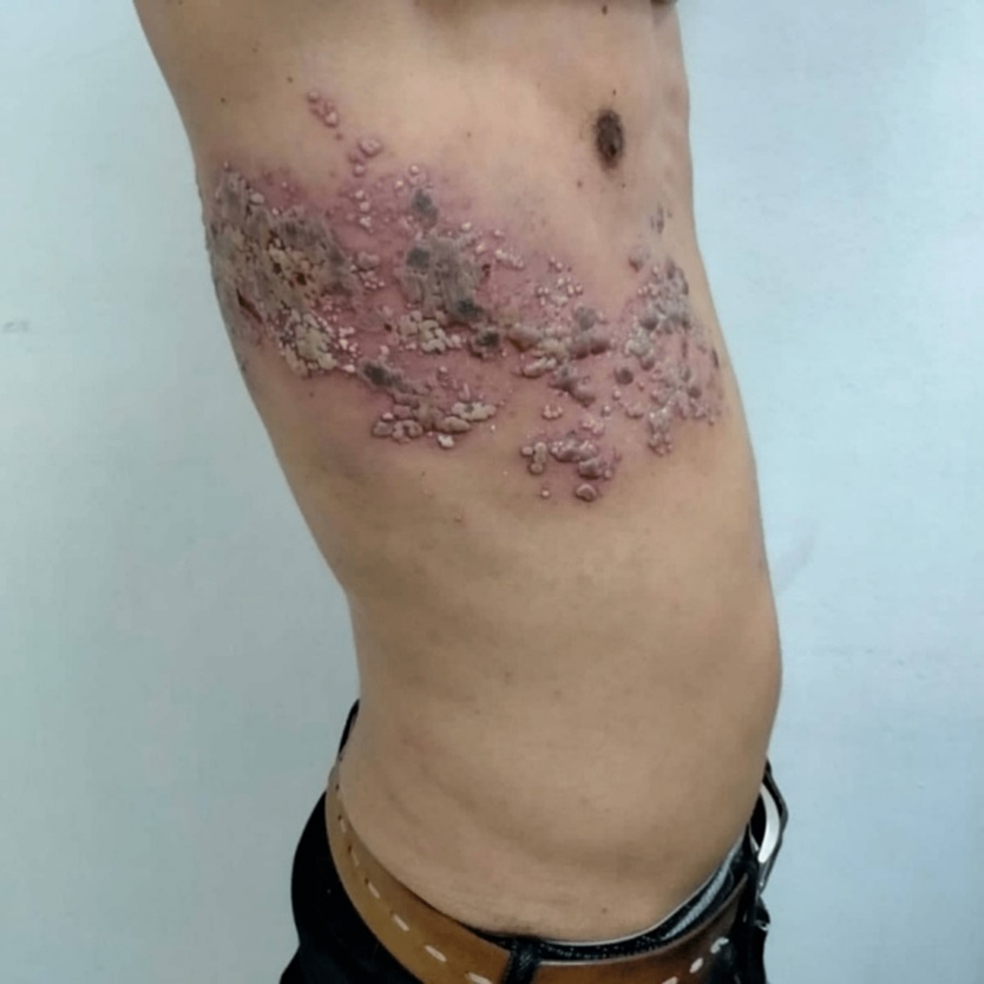
Intercostal herpes zoster in a young renal transplant patient.

**Figure 2:**
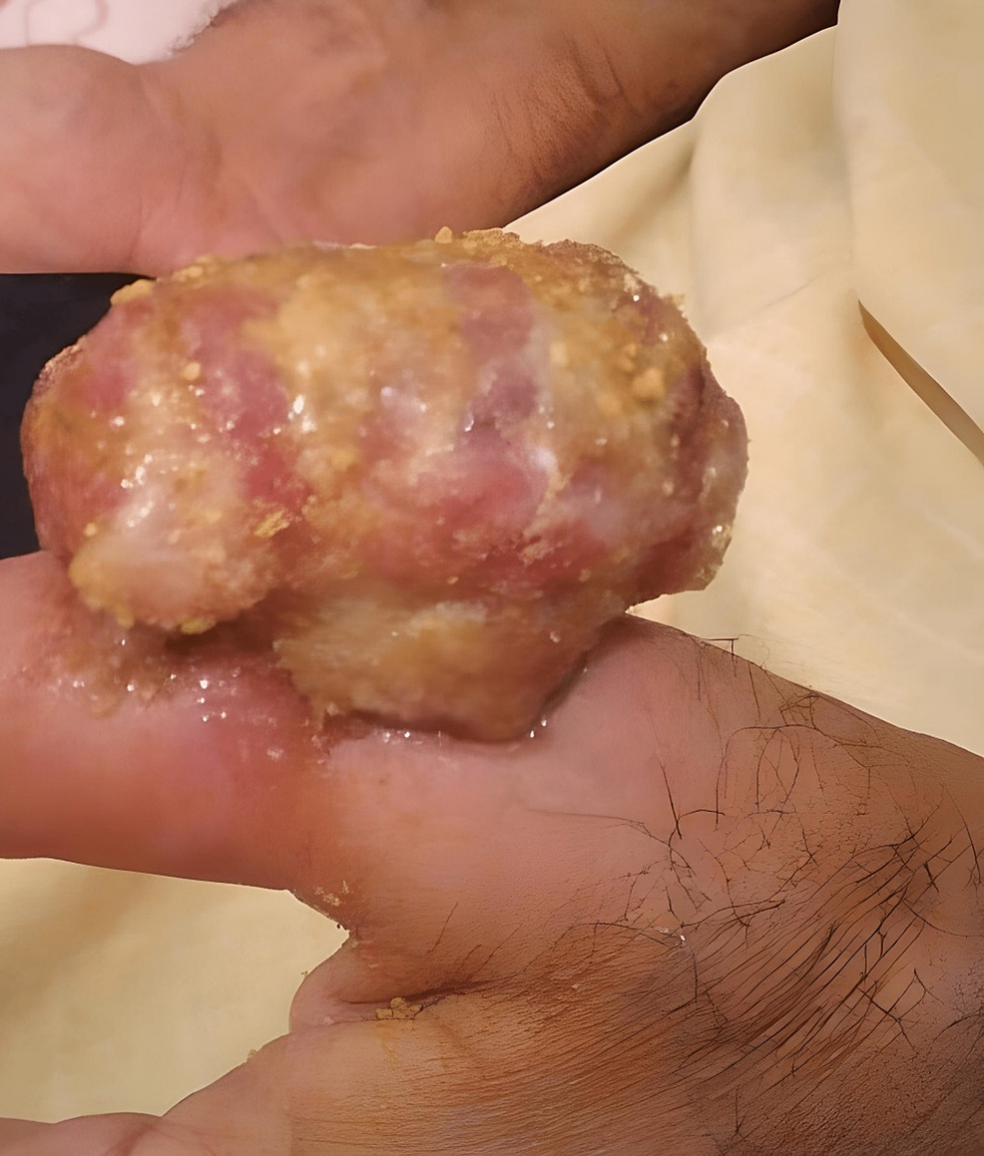
Atypical mycobacteriosis on the back of the thumb in a renal transplant recipient.

**Figure 3:**
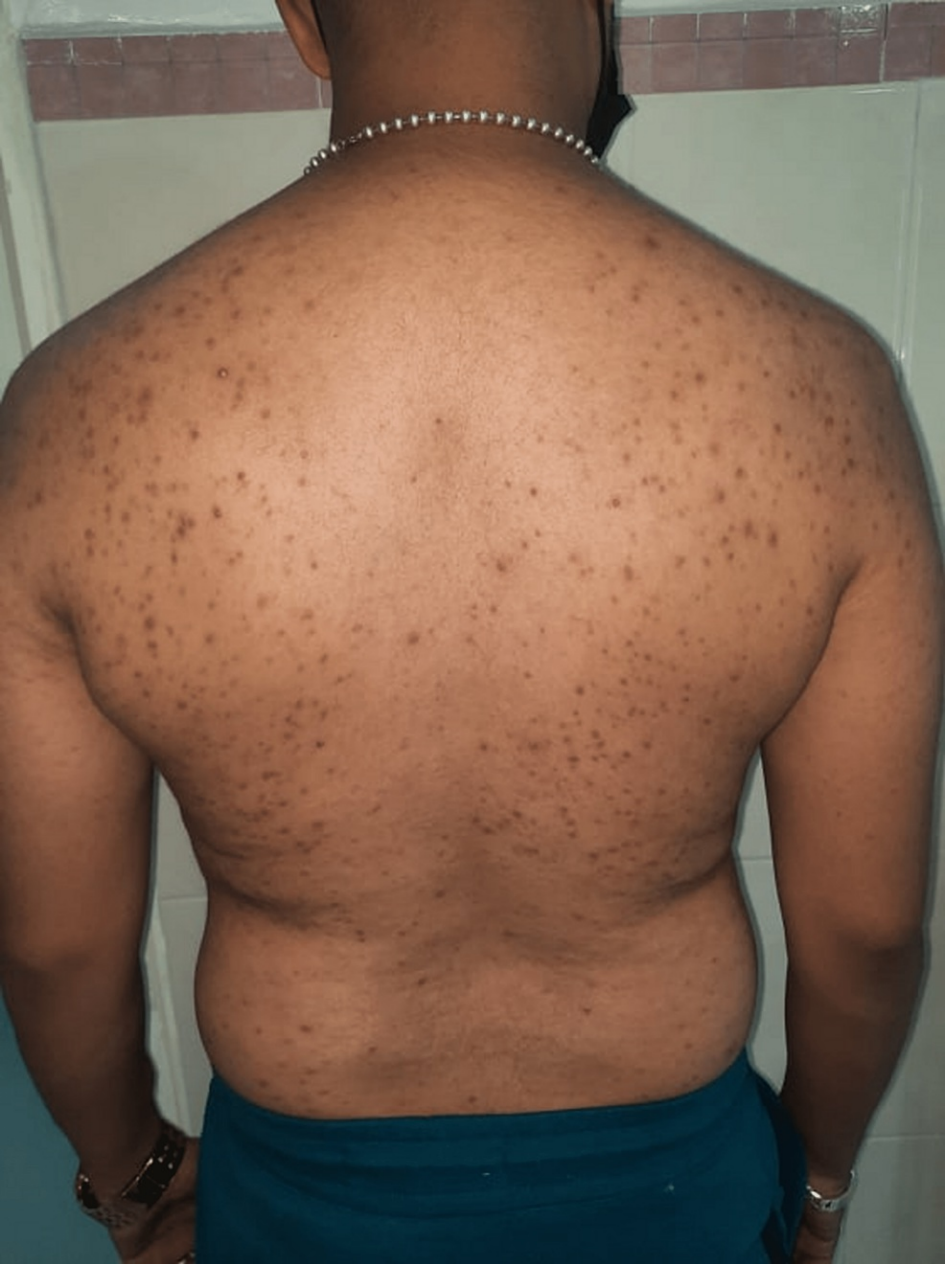
Acneiform lesions in a young renal transplant recipient.

The mucocutaneous complications observed in kidney transplant patients are represented in table 1 (Figure 1-3).

**Table 1:** Distribution of mucocutaneous manifestations in kidney transplant patients.

Infectious complications (53.4%)						Inflammatory complications (36.6%)		Other complications (10%)	
Fungal (26.8%)		Viral (20%)		Bacterial (6.6%)					
Epidermomycosis	n=2	Warts	n=3	Atypical mycobacteriosis (Figure 2)	n=1	Acne (Figure 3)	n=4	Actinic keratosis	n=1
Onychomycosis	n=2	Herpes labialis	n=2	Boils	n=1	Urticaria	n=3	Cutaneous xerosis	n=1
Seborrheic dermatitis	n=2	Intercostal herpes zoster (Figure 1)	n=1			Rosacea	n=1	Bruises	n=1
						Simple maculopapular exanthema	n=1		
Oral candidiasis	n=1					Aphtae	n=1		
Pytiriasis versicolor	n=1					Black hairy tongue	n=1		

**Figure 4:**
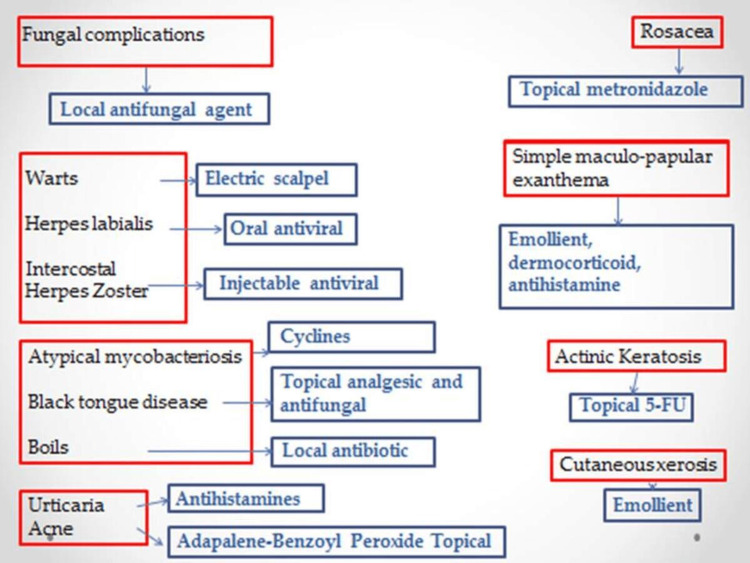
Management of mucocutaneous complications in renal transplant recipients.

Three patients had a mycological sample (10%), six patients had a bacteriological sample (20%), and two patients had a cutaneous biopsy (6.66%). A pharmacovigilance investigation was carried out on the patient presenting a simple maculopapular exanthema, whose imputability score incriminated the enzyme conversion inhibitors. Treatment was symptomatic with a good evolution in all the patients (figure 4). All the patients were under photoprotection. The immunosuppressive treatment (Mycophenolate Mofetil) was temporarily reduced in the patient presenting with intercostal herpes zoster.

After statistical analysis, mucocutaneous complications in our study were significantly associated with advanced age (p = 0.039), male gender (p = 0.025), anemia (p < 0.001), HLA non-identical donor (p = 0.001), and with the use of Thymoglobulin or Tacrolimus (p < 0.001). However, the use of Ciclosporin or Basiliximab was considered a protective factor (p < 0.001) (Table 2).

**Table 2:** Association between the different factors and the occurrence of mucocutaneous complications in kidney transplant patients.

Factor		Skin manifestation+ (n=30)	Skin manifestation – (n= 56)	Relative Risk	Confiance interval 95%	p value
Age		42.73	36,71	-	-	0.039
Gender	Female	8	29	1.42	[1.04-1.92]	0.025
	Male	22	27			
Diabetes		8	7	1.47	[0.84-2.59]	0.099
Arterial hypertension		15	19	1.27	[0.90-1.79]	0.146
Heart disease		4	0	0.31	[0.23-0.43]	0.013
Obesity		8	5	1.81	[0.89-3.67]	0.061
Anemia		15	7	2.40	[1.28-4.50]	<0.001
HLA	Identical	10	40	1.8	[1.21-2.66]	0.001
	Non-identical	20	16			
Median age of renal transplantation (Year)		4.97	4.32	-	-	0.444
Tacrolimus		23	7	3.75	[1.94-7.22]	<0.001
Ciclosporin		7	50	0.23	[0.11-0.48]	<0.001
Thymoglobulin		20	16	2.40	[1.28-4.50]	<0.001
Basiliximab		10	40	0.11	[0.13-0.54]	<0.001

## Discussion

Only a few studies described mucocutaneous complications in kidney transplant patients. Four studies described general skin complications [5-8], while two others only focused on tumors in all solid organ transplant recipients [9-10]. Other studies described general infectious manifestations in kidney transplant patients [11-13]. During our study period, thirty cases of mucocutaneous complications were identified. This incidence remains low compared to other studies, however, the frequency among the total number of transplant recipients (30/86) is on the same line as that of Savoia [7], higher than that of Navarro [5] and lower than that of Scalbert [8] (Table 3).

**Table 3:** Comparison between the different series of mucocutaneous complications in kidney transplant recipients.

Series	Incidence of mucocutaneous complications among transplant recipients	Period	Mean age (years) / Gender	Clinical manifestations
Navarro et al., 2008 [5]	112 / 1017 (11%)	1979-2007	61 /Men++	Skin cancers
Chen et al., 2010 [6]	143 / -	2006-2009	50 / Men++ (60.8%)	Infectious manifestations 17.27%
				Drug toxicity 16.82%
				Skin cancers 7.27%
				Others 58.63%
Savoia et al., 2011 [7]	99 / 282 (35.1%)	2009-2016	60 / Men++ (62%)	Infectious manifestations 16.7%
				Skin cancers 35.1%
				Drug toxicity 10.63%
				Others 37.57%
Scalbert-Sadones, 2015 [8]	170 / 266 (64%)	2012-2014	55.1 / Men++ (64%)	Infectious manifestations 34%
				Skin cancers 28%
				Inflammatory manifestations 20%
				Others 18%
Our series	30 / 86 (34.88%)	2020-2021	42.73 / Men++ (73%)	Infectious manifestations 53.4%
				Inflammatory manifestations 36.6%
				Precancerous lesions 3.3%
				Others 6.6%

The risk factors of mucocutaneous complications in kidney transplant patients aren’t clearly defined. We found that advanced age and male gender were risk factors, which is in the same line as Navarro and Garett's data regarding skin cancers in transplant recipients [5,10]. Although diabetes is known to be a risk factor for infection [14], its association with the occurrence of mucocutaneous complications wasn't significant, which is consistent with the data of Gras [12]. However, anemia was considered a risk factor, which can be explained by hypoxia and altered neutrophil function. The non-identical HLA between donor and recipient was also considered a risk factor, which may be explained by the administration of a high dose of immunosuppressants in these patients given the high risk of rejection.

Immunosuppressive therapy used during kidney transplantation can lead to skin complications by specific toxicity or by immunosuppression that can be life-threatening [15]. Azathioprine has a pro-carcinogenic action [16]. This wasn't used in our series which may explain the absence of tumors. Mycophenolate Mofetil, received by all our patients, mainly causes aphthoid lesions, acneiform eruptions, and nail damage. Corticosteroids, also received by all our patients, can cause skin atrophy, purpuric lesions, acneiform eruptions, and rosacea. Ciclosporin’s skin toxicity is dominated by gingival hyperplasia, hypertrichosis, acneiform lesions, and xerosis, while that of Tacrolimus is reversible alopecia [17]. In Gras’s study, more patients who developed tuberculosis received Thymoglobulin [12]. However, Basiliximab was a protective factor in Chen’s study [18], which is consistent with our data.

Our series is characterized by the predominance of infectious complications, given the short time frame of kidney transplants, during which infectious complications predominate [19]. This high prevalence could also be explained by the non-use of mTOR inhibitors known for their anti-infective effect [20]. Inflammatory manifestations are mainly related to drug toxicity cited in the previous paragraph. Their high prevalence in our series can be explained by the young age of our patients predisposing them to develop acne and urticaria. The absence of tumors could be explained by regular monitoring (including Mycophenolate Mofetil blood dosage), photoprotection, and the short post-transplant delay. Tumors usually appear many years after the kidney transplant [21].

Treatment of mucocutaneous complications in renal transplant patients is the same as in the general population, with a preference for local treatments to avoid drug interactions. Nevertheless, manifestations with vital or functional prognosis must be treated with an oral or venous route, sometimes requiring a reduction in the dose of immunosuppressants. Drug dosage must be adapted to the renal function. 

Recommendation

**Figure 5:**
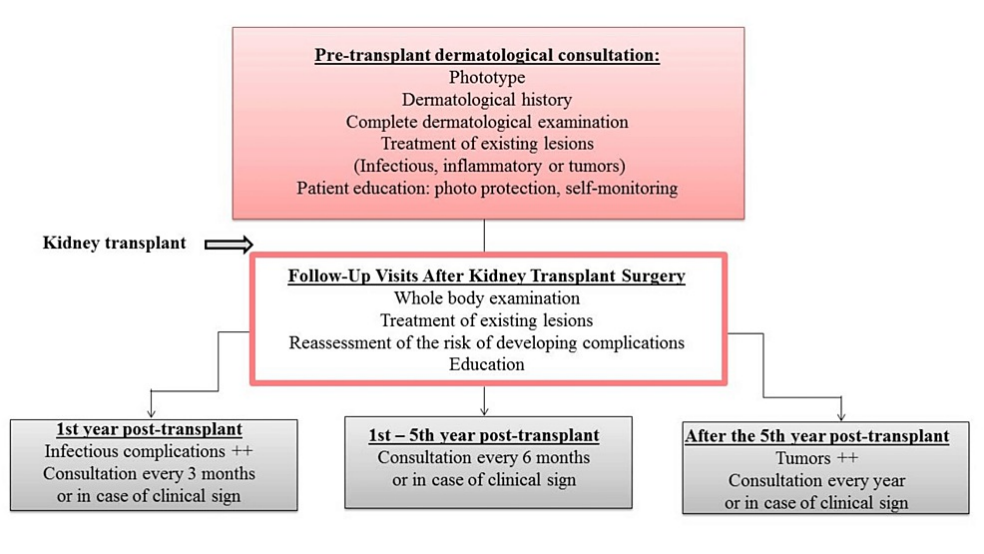
Follow-up of kidney transplant patients.

A monitoring scheme is proposed in figure 5. 

## Conclusions

Immunosuppressive therapy in renal transplant patients can lead to dermatological complications either by drug toxicity or by immunosuppression. they can have dramatic functional and life-threatening consequences, hence the importance of strict monitoring of these patients. Dermatological monitoring of kidney transplant patients requires collaboration between the dermatologist and the nephrologist in order to anticipate the onset of mucocutaneous complications and treat them early. It is also necessary to educate the patient about the preventive measures and the interest in an early consultation at the slightest sign.
